# Dr. Whelpley

**Published:** 1892-07

**Authors:** 


Dr. Henry M. Whelpley was married to Miss Laura Eugenie Spannagel, on June 29th, in St. Louis. Dr. and Mrs. Whelpley will be at home after August 15, 2342 Albion Place, St. Louis, Mo.

